# Open access: the view of the Public Library of Science

**DOI:** 10.1111/j.1538-7836.2006.02008.x

**Published:** 2006-07-01

**Authors:** V BARBOUR, M PATTERSON

**Affiliations:** Public Library of Science, European Office Cambridge, UK

## Abstract

*See also* Robinson A. Open access: the view of a commercial publisher. This issue, pp 1454–60.

‘I want a poor student to have the same means of indulging his learned curiosity, of following his rational pursuits, of consulting the same authorities, of fathoming the most intricate inquiry as the richest man in the kingdom’. Sir Antonio Panizzi, Principal Librarian of the British Museum, 1836.

Open access (OA) to scientific and medical literature is not a new idea. By transforming policy and practice at the British Museum Library, Antonio Panizzi took significant steps towards this goal back in the 19th century. Regarded as one of the greatest librarians of his time, were he alive today, it is hard to imagine that Panizzi would not be embracing the potential of the Internet to finally achieve his vision of free and equitable access to scholarship.

At around the same time as Panizzi's pioneering work, the world of medical journals was also flourishing. These journals were becoming established as the mechanism for the registration and validation of ideas through peer-review, and as vehicles for the sharing of this information. Many of these journals still exist today, but for most of the past few hundred years they have only been available on paper, and distributed by the currently available modes of transport. Distribution was therefore an expensive undertaking and publishers have traditionally recovered these costs by charging a fee to read the journals.

An important concept, which has been central to the business of publishing and distributing information on paper, is copyright [1]. Initially introduced several hundred years ago to protect the rights of the printers themselves from being undercut by cheaper imitators, the notion of copyright was later extended to serve the rights of creators and authors of works, so that they could make a living from their efforts. Unlike the authors of novels, however, authors of articles in scholarly journals are not concerned about their rights to royalties, and the usual practice has therefore been that the author transfers their copyright, or an exclusive right to distribution, to the publishers. As the overall aggregators of the information in journals, it was argued that the publishers would be best placed to protect authors from infringements such as plagiarism.

The scholarly journals of today are therefore the product of policies and practices that have evolved over several hundred years of printing on paper. For most scientific journals the published work is written by one group of scientists, peer-reviewed by a second set, and frequently edited and collated by a third set. These scholars are rarely paid by the publisher, and yet it is the publisher that has the exclusive right to distribute the work. To cover the costs of the dissemination of this information, publishers charge for access to the journal, by the article, by the issue, or more frequently by an annual subscription to a journal. Gradually, the idea of subscription-based publishing has become the norm for scientific publishers. Such journals have been very successful; over the past few decades in particular they have proliferated at a tremendous rate, and at the same time subscription journal publishing has emerged as a highly profitable business. But times are changing: the Internet has given rise to an entirely different approach to the dissemination of research results.

## How does the Internet change things?

When the Internet first appeared, few understood how it might change the dissemination of the literature. For scientific publishing it is, however, nothing less than a revolutionary technology. Amongst the powerful new capabilities that the Internet offers are the following:

1. Information no longer has to be disseminated on paper. Even if the reader ultimately generates a paper copy, the printing of the work itself is not a limiting factor in its dissemination. Dissemination by means of the Internet is therefore cheap and very fast, compared with the cost of printing and mailing bulky paper journals.
2. The Internet is global, and so information can be disseminated much more broadly than is possible in print.
3. The vast storage space of the Internet also means that it is possible to allow access not only to the results of papers, but also to relevant raw data and background information, which greatly increases their value for scientific research.
4. The Internet can be searched. Google™, for example, have been pioneers in developing mechanisms for indexing and retrieval, such that it is now possible for anyone to find and retrieve information of interest to them from computers all over the planet.

Quite suddenly then, the traditional means of disseminating information has a potent alternative. By publishing research articles on the Internet, it is now possible to provide virtually unlimited access to that information, and thus to take a massive step towards achieving the vision of Antonio Panizzi. The goal that becomes possible with the Internet has been termed ‘open access’ [2].

## What is open access?

Although the term open access has been used in different ways, its two most important attributes are as follows:

1. When work is published, it is immediately and freely available to anyone with an internet connection.
2. The author retains copyright, but licenses anyone to read, download, copy, redistribute and use the article for any legal purpose, as long as the author is properly acknowledged as the creator of the work.

The second component of OA is very important, because it makes possible any number of potential re-uses of research literature, which are severely hampered today by the restrictive licensing arrangements adopted by most publishers. By contrast, the license that is used by OA publishers such as the Public Library of Science (PLoS) and Biomed Central was devised by the Creative Commons and is called the attribution license [3]. This license is also now being adopted by several other publishers offering OA options to authors.

## Open access: who needs it?

OA maximizes access to the literature, but goes one step further by maximizing the utility of the literature as well. The benefits of OA are therefore far-reaching, and we can only anticipate a fraction of these. The authors of papers, for example, will be reaching the broadest possible audience, and so their work is more likely to be read and cited, and will in all senses have greater impact than if it were published in a journal with a restricted readership.

The scholarly readers of journals will also benefit, because they will have access to any research literature of relevance to them. Importantly, researchers everywhere, from human immunodeficiency virus physicians in Africa to geneticists in New York, will be able to access the literature, although it is also important to acknowledge that the availability of Internet connectivity will continue to hamper access even to OA material in the poorest parts of our planet.

The research community will also be able to interrogate, navigate and mine the literature in entirely new ways. Indeed, the public online availability of biological data, such as the human genome sequence, has been another of the inspirations for open access to literature. The free availability of these data has spawned an entirely new field of bioinformatics, which has led to the development of new resources and tools to use the data. One can only begin to imagine the types of tools for knowledge discovery that could be devised by the burgeoning text-mining community if the entire body of scientific and medical research were publicly available online.

Beyond the scholarly community, the Internet has also made apparent the interest of those who are not professional researchers in medical and scientific literature. This diverse group includes teachers and their students, practicing physicians and patients, historians and politicians, and many more besides. Although some commentators have asserted that the public will not benefit from the oftentimes arcane or esoteric information that is published in specialized journals, there are many who will. For example, in their report on scientific publishing in 2004, the UK House of Commons Science and Technology Committee [4] concluded:

‘It is not for either publishers or academics to decide who should, and who should not, be allowed to read scientific journal articles. We are encouraged by the growing interest in research findings shown by the public. It is in society's interest that public understanding of science should increase. Increased public access to research findings should be encouraged by publishers, academics and Government alike’.

## How do you pay for OA?

Although free online access to research literature is a highly desirable and achievable goal, publishers are struggling to identify a business model that compares with charging subscription fees. There has been substantial experimentation with the bundling of content in the online medium, and the creation of new licenses, but the essence of the transaction has remained the same: content is available only to those who have paid for it.

Nevertheless, a business model to support OA is emerging, which turns the subscription model on its head. Instead of paying to read the literature, there is a payment to publish. If a publisher can recover the full costs of publishing via a publication fee, and other sources of revenue such as advertising, there need be no charges to the reader. Thus the literature can be made freely available on the Internet, for anyone to read and use.

The publication-fee approach has been adopted by OA publishers and is now offered as an option by several major traditional publishers such as Blackwell Publishing, Oxford University Press and Springer. The key to the success of this model is that funding agencies regard publication as an integral part of the research process, and therefore include in research grants funds to cover the OA publication fees. Increasingly, the funding agencies are recognizing that OA is also in their own interests, because it maximizes the impact of the research that they fund.

## Obstacles to open access

Given the opportunities afforded by the Internet, and the social and scientific advantages of OA, it is reasonable to ask why OA has not been more readily adopted. The answer to this question is a mix of financial concerns, resistance to changing the status quo, and a lack of appreciation of the limitations of subscription journals relative to the powerful benefits of OA, amongst many of the key stakeholders in scientific publishing.

One concern often expressed is that not all authors will be able to pay the publishing fee, that is, we might end up changing the publishing system such that those who cannot currently afford access to the literature become those who cannot afford to publish their findings. This concern will be overcome by ensuring that researchers have access to funds in their research grants to cover publication fees (provided by funding agencies or institutions), or in cases where no such funds exist, by fee waivers from publishers. PLoS and Biomed Central, for example, both offer fee waivers to authors with insufficient funds so that lack of funds is never a barrier to publication. It is also crucial that for peer-reviewed journals, decisions on publication are completely independent of authors’ ability to pay: journals such as those operated by PLoS have set up mechanisms so that editors and reviewers have no access to information on authors’ ability to pay.

There are challenges too for funding agencies. OA requires that they provide additional funds to authors to cover OA fees. Agencies such as the Wellcome Trust, however, have calculated that the costs of publishing are very small relative to the costs of funding research itself. They have also conducted their own survey of the publishing landscape and concluded that the overall costs of publishing, that is, to society as a whole, will become much clearer, and are probably smaller than in the current system [5]. Many other funding agencies have now added their support to the concept of OA publishing, as indicated by the signatories of the Berlin Declaration on Open Access [6] who now include most of the major funding agencies in Europe.

Another group of publishing stakeholders who will be affected by OA are scientific societies, many of whom have benefited financially from the income from subscription journals. Scholarly societies put that money to good use within their respective communities and perform important functions for scientific and medical research as a whole. Many have expressed concern that a shift towards OA would reduce their income and limit the good works that they are able to perform. Nevertheless, maximizing access to the research of their community also lies at the heart of the mission of many societies and there are already examples of societies who are actively supporting OA. *PLoS Computational Biology*, for example, is being published in collaboration with The International Society for Computational Biology.

A further group who are important in driving the transition towards OA, but who have not previously been considered as important players in scientific publishing, are patients and their advocates. In the USA in particular, the Alliance for Taxpayer Access has brought this issue to the highest levels of government [7].

OA is only a few years old, but it already seems clear that it is here to stay. There are some important concerns about how to negotiate the transition from subscription-based journals, but the obstacles are being overcome. Nonetheless, given its short life so far, the financial model of OA remains as yet unproven, and financial viability is the goal toward which all OA publishers are now striving.

## The open access publishing landscape

### Where we are now?

In 2006, the publishing landscape contains an increasing number of fully open-access journals [8]. There are also many journals that are experimenting with hybrid models, offering OA models to authors who have the funds to pay for this. In addition, many academic institutions are beginning to host their own electronic repositories of research output [9]. These public repositories would ideally hold the final published versions of articles, but in the face of some opposition from publishers, researchers are often only able to deposit accepted but unedited versions of their articles. This has led to some concern about different versions of manuscripts being present in the public domain. Nevertheless, the repositories are adding an important channel for public access to scholarly output.

### Where will we be in 10 years?

It is almost inconceivable that in 10 years’ time, OA to the primary research literature will not have become the favored model of publishing. The challenge for all with an interest in publishing is to work out the way to get there. Any solution must recognize the fears of current publishers and societies over possible loss of revenue, while at the same time not allow the profitability of the existing model to stifle innovation. Another issue that will need to be addressed is the long-term sustainability of the digital archives currently being created. There might well be some major changes in the way that journals look and ‘feel’ online, and in the way that scholarship is disseminated and communicated online. However, there can be little doubt that OA to this information will be to the long-term benefit of authors, readers, and ultimately society.

### Why should the medical profession care about open access?

On the one hand, doctors want access to the research literature, but on the other hand they may be wary of their patients having access to it; doctors can feel somewhat threatened by over-informed patients. But, as hinted at above, there is no way back. Certainly, patients armed with information from peer-reviewed medical articles are much better able to participate constructively in their healthcare than those who come up with only rather dubious secondary information, that has not been peer-reviewed, from a Google™ search. Nonetheless, when PLoS launched *PLoS Medicine*, we were concerned that doctors might see OA publishing as a threat. So, we did a poll of doctors on the UK medical register via http://www.doctors.net.uk, a medical website. What we found was that, of the 2329 doctors who responded, 67% were in favor of their patients having access to the peer-reviewed literature, and the doctors wanted access too.

Now that authors have an increasing number of OA options, authors themselves have a tremendous opportunity to drive this change, so that users of the medical literature can fully benefit from open access to research and scholarship. We end with the words of the Stanford Lane Medical Library [10], who sum the issues up as follows:

‘Open Access: Where You Publish Makes A Difference Each author's choice of where to publish adds another brick to a complex publishing structure. Your choice may have a dramatic effect on how accessible, or inaccessible, your research is. Your decision can limit or facilitate others’ digital access to significant research. Familiarizing yourself with the raging controversy over the new Open Access publishing model will help you make informed decisions about the impact of your choice on where to publish – on your professional standing, on library budgets, and ultimately on scholarship itself. Finding a balance is challenging. The stakes are high for all’.
